# A Design of Experiments Approach to Identify Critical Processing Parameters for Manufacture of an Autologous Platelet Gel for Diabetic Foot Ulcer

**DOI:** 10.3390/pharmaceutics17111482

**Published:** 2025-11-17

**Authors:** Aleksandra Olszewska, Olga Egorova, Gabriella Gaggia, Kalliopi Mylona, Simon Pitchford, James Rickard, Ben Forbes

**Affiliations:** 1 Institute of Pharmaceutical Science, King’s College London, Franklin-Wilkins Building, Stamford Street, London SE1 9NH, UK; 2 Department of Mathematics, King’s College London, Strand Building, Strand Campus, Strand, London WC2R 2LS, UK; 3 Biotherapy Services Ltd., The Clarence Centre for Enterprise & Innovation, 6 St. George’s Circus, London SE1 6FE, UK

**Keywords:** RAPID^TM^ Biodynamic Haematogel, quality assurance, design of experiments, quality by design

## Abstract

**Background/Objectives:** RAPID^TM^ Biodynamic Haematogel is a platelet-based gel for wound healing in diabetic foot ulcers. This study aimed to identify the processing parameters that impact on the quality of this autologous point-of-care manufactured product. **Methods:** An innovative design of experiments (DOE) approach utilizing a split-plot factorial design and linear mixed-effects models enabled the evaluation of six processing parameters on time to gel and the exudation of gel releasate. **Results:** Across all manufacturing conditions, time to gel was 181.3 ± 179.2 s (n = 28) and the total mass of releasate exuded in 2 h was 5.6 ± 2.1 g (n = 28). Two processing parameters (temperature 15–30 °C and pre-mixing of ascorbic acid and L-PRP) had a significant impact on releasate exudation and/or time to gel. The other processing parameters (time-to-thrombin use, mixing time, WBC content and filtering of the thrombin) had little effect. The amount of releasate exuded was affected by the interaction of the temperature and time-to-thrombin use. Time to gel was affected by the mixing time and by pre-mixing the ascorbic acid and L-PRP in conjunction with temperature. **Conclusions:** This study illustrates an optimization of DOE methodology to inform pharmaceutical product development and identify factors that influence variability in the RAPID Biodynamic Haematogel product.

## 1. Introduction

Hard-to-heal chronic wounds burden healthcare systems worldwide. In the UK, the NHS spent GBP 8.3 billion on wound management in 2017/18 [1]. A significant proportion of this spending was for chronic ulcers of which diabetic foot ulcers (DFUs) are one of the most prominent. It has been estimated that around 19–34% of all diabetic patients will develop a DFU at some point during their life [2,3]. The increasing prevalence of diabetes together with a lack of successful long-term treatments for DFUs brings an urgent need to develop accessible, effective treatments.

Platelet-rich plasma (PRP) products, such as the RAPID Biodynamic Haematogel, are a novel treatment modality for various indications including topical treatment for DFUs. Rich in crucial growth factors, PRP and its gel form have been shown to aid wound healing [4]. Autologous PRP gels are prepared at the point of care (POC) and often exhibit high batch-to-batch variability due to the heterogeneity of the starting material (individual patient’s blood) and variation in manufacturing processes. Consequently, control of PRP gel manufacturing using Good Manufacturing Practice (GMP) is a critical part of quality assurance and can be achieved by adopting a quality by design approach in line with ICH Q8 [5].

In this study, RAPID Biodynamic Haematogel, a leukocyte and platelet-rich plasma (L-PRP) product, was investigated to identify putative critical processing parameters (cPPs) in gel manufacture that might require control to ensure product quality. Processing parameters that may have an impact on the final product attributes included white blood cell (WBC) content (% hematocrit) and temperature, along with filtration, time-to-thrombin use and mixing variables [5,6,7,8]. The effects of these processing parameters on two gel attributes that are reflective of gel quality and consistency were measured, (i) time to gel and (ii) exudation of releasate from the gel. A state-of-the-art design of experiments (DoE) methodology was applied to efficiently evaluate the influence of multiple cPPs on product quality [8]. While DoE has been widely applied in pharmaceutical manufacturing and bioprocess optimization [9,10], its application in POC autologous product development is novel.

The bespoke split-plot statistical model developed to satisfy the inferential aims of the study was an advanced multi-objective design to enable efficient handling of hierarchical variability in donor characteristics and the practical need to group experimental runs that utilized the same temperature. The model implements restricted randomization to address the challenge of analyzing both fixed effects (processing parameters, e.g., temperature, mixing time) and random effects (e.g., donor-to-donor variability). This dual-level approach provides robust insights into cPPs while effectively addressing the constraints and limitations typical for POC settings. Corresponding statistical analyses involved fitting linear mixed-effects models for each of the responses, thus providing an interpretable inference on the relationship between the fixed experimental factors (processing parameters) and the response, as well as estimates of the between-participant and within-participant (blood donor) uncertainty measures [11]. Within-donor and between-donor variability is often overlooked in PRP research, and this approach aligns with the principles of personalized medicine, offering greater precision in determining cPPs and establishing working ranges to enhance product uniformity. Moreover, it addresses a critical gap in the literature by providing a framework for optimizing autologous product manufacturing in decentralized clinical settings. Any processing parameter identified as critical in these experiments can be controlled by proposing working ranges that will improve gel uniformity in the clinic [12].

We have previously reported the manufacturing process to produce the RAPID Biodynamic Haematogel and described the product characteristics [13]. In that study, we described in detail the point-of-care manufacturing protocol and analyzed the cellular and biochemical composition of the gels formed. Notable variability between preparations was observed in platelet and leukocyte concentration, time to gel, the temporal profile of liquid exudation from the gels and the content of growth factors VEGF and PDGF in the releasate. Building on this foundation, the present study was designed to investigate the effect of putative cPPs on the uniformity of the gels formed as a means of controlling product quality. The influence of six factors (temperature, mixing time, WBC content, time-to-thrombin use, filtration of the thrombin, pre-mixing of reagents) on output variables (time to gel and the amount of releasate exuded by the gel in 2 h) was explored to evaluate the criticality of the processing parameters on these gel qualities. The tailored split-plot design used not only enhances experimental efficiency but also provides a robust means of understanding cPP criticality. This integration of advanced DoE methodologies and mixed-effects modeling was used to identify the manufacturing steps that require control to ensure consistency and quality for RAPID Biodynamic Haematogel manufacture at POC in clinical environments.

## 2. Materials and Methods

### 2.1. Fresh Human Blood

Human whole blood in acid citrate dextrose (ACD) solution from healthy donors was sourced from Cambridge Bioscience (Research Donors, Cambridge Bioscience, Cambridge, UK). Experiments were conducted over 8 days with 260 mL of fresh human blood from a single donor processed in 60 mL increments to provide 4 experimental runs on each day. Platelet and leukocyte counts were performed on each batch of blood used for experiments and on the L-PRP generated during the manufacture of each gel. Blood or L-PRP was diluted 1:100 with Stromatol solution (Stromatolytic agent for blood platelet counts, Mascia Brunelli, Milan, Italy) and cells were counted manually using a hemocytometer (Neubauer improved counting chamber, Paul Marienfeld, Lauda-Königshofen, Germany). All procedures adhered to the regulations outlined in the Human Tissue Act 2004.

### 2.2. Standard Protocol for RAPID Biodynamic Haematogel Manufacture

The standard Biotherapy Services POC RAPID manufacturing protocol (patent EP3749336) was followed to generate the RAPID leukocyte and platelet-rich plasma (L-PRP) gels [13]. Briefly, the manufacturing process consists of three steps. Firstly, whole blood was processed in 60 mL increments using an Arthrex Angel System centrifuge (Arthrex, Munich, Germany) with a pre-programmed two-step centrifugation protocol. There are two available presets to prepare the RAPID acute (8% hematocrit) and chronic (2% hematocrit). The device’s hematocrit (Hct) setting refers to the % of red cells intentionally allowed into the L-PRP product. We used two presets: 8% Hct (‘acute’), which collects deeper into the buffy coat/RBC interface to yield a larger, more leukocyte-rich L-PRP, and 2% Hct (‘chronic’), which restricts RBC carryover and typically produces a ‘purer’ L-PRP with fewer leukocytes. The two-step centrifugation incorporates a fast and slow spin (3500 RPM for 2:56 min followed by 3000 RPM for 8:32 min). Centrifugation separated the blood into two fractions: a low-volume L-PRP and a larger volume of platelet-poor plasma (PPP). Following blood separation, the L-PRP fraction was diluted with PPP to 6 mL. The remaining PPP was used to generate autologous thrombin-rich serum by using an Arthrex Thrombinator^TM^ System (Arthrex, Munich, Germany). The time to form the clot within the device, i.e., thrombin-rich serum generation time, was measured using a stopwatch. Finally, to form the RAPID gel, the 6 mL L-PRP was mixed with 0.75 mL of ascorbic acid (100 mg/mL, Sanorell Pharma, Bühl /Baden, Germany) in a small plastic cup, together with 2 mL of thrombin-rich serum to activate platelets and the coagulation cascade. The mixture was swirled gently, and the time taken for the gel to form was recorded. Each RAPID gel was weighed at the point of manufacture.

### 2.3. Development of a Design of Experiments (DoE) Approach

**Figure 1:**
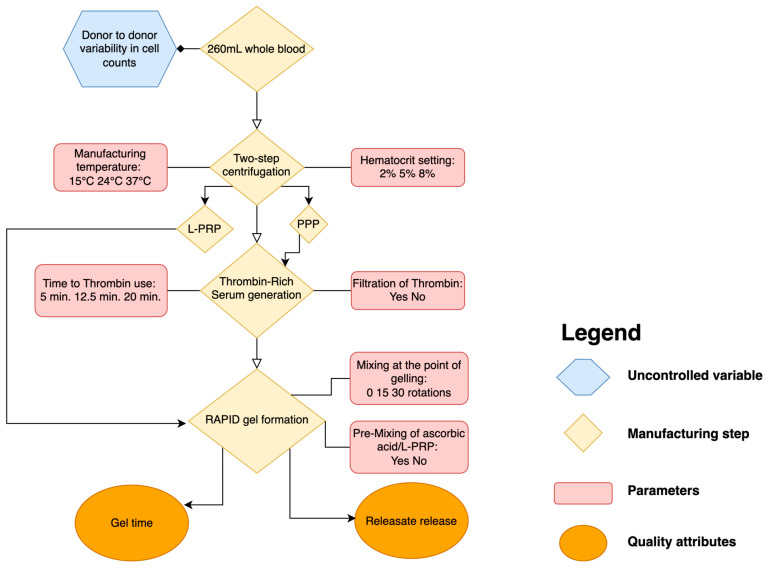
**Simplified flowchart representing the RAPID Biodynamic Haematogel manufacturing process.** Whole blood undergoes a two-step centrifugation process to yield leukocyte and platelet-rich plasma (L-PRP) and platelet-poor plasma (PPP), from which thrombin-rich serum is generated. The final RAPID™ gel is formed by combining L-PRP, thrombin-rich serum and ascorbic acid. Key process parameters (e.g., temperature, hematocrit, time-to-thrombin use), manufacturing steps and uncontrolled donor-specific variables are highlighted. The diagram also illustrates the quality attributes.

Six processing parameters were investigated to evaluate their impact on gel formation and the exudation of releasate from the gel (Table 1; Figure 1).

The processing parameters that were studied in the DoE are highlighted in red and their levels defined (Figure 1). Quality attributes are highlighted in orange. The experiment was designed to accommodate four continuous and two categorical variables, plus the logistics of conducting the experiment.

To make meaningful inference on how processing parameters affect the outcomes of the process, a response surface approach was used to fit a full quadratic polynomial model for each of the continuous outputs [14]. The experiment was carried out over 8 days, with blood from a different donor being used on each day for 4 experimental runs (single iterations of the process) of gel manufacturing utilizing different combinations of PP variables. It proved unfeasible logistically to vary the manufacturing temperature across these runs, and the experiment was designed with the temperature held constant within each of the days, while the 5 other factors (Table 1) varied across the runs. Such a restricted randomization structure is known as a “split-plot” experiment, where some factors (manufacturing temperature in this case) are assigned to homogenous larger experimental units (days of experiment/donor) and others are assigned to nested units within these (runs). The corresponding linear mixed-effects model for a continuous outcome was formulated as follows:

(1) $$Y=Z\varepsilon_{0}+X\beta \;+\;\varepsilon$$

where Y is the output vector of length n, matrix Z with $n_{d}$ columns containing day/donor indicators for each of the n runs, $\varepsilon_{0}$ and ε are between- and within-day/donor error terms. Both error terms are assumed to be following normal distributions with zero means and constant variances $\sigma_{0}^{2}$ and $\sigma^{2}.\;X$ multiplied by vector β represents the regression model (fixed effects) on the controlled process parameters (Table 1).

For an individual sample i (i = 1…4) within day/donor j (j = 1…8), it can be rewritten as follows:

(2) $$y_{ij}=\;\beta_{0}+\beta_{MT}X_{MT}^{\left(j\right)}+\beta_{t}X_{t}^{\left(i,j\right)}+\beta_{W}X_{W}^{\left(i,j\right)}+\ldots +\beta_{t\times W}X_{t}^{\left(i,j\right)}X_{W}^{\left(i,j\right)}+\varepsilon_{0}^{\left(j\right)}+\varepsilon {,}^{\left(i,j\right)}$$

With the maximum set of individual fixed effects comprising linear terms, two-factor interactions and quadratic terms of the factors that are listed in Table 1; note that the values of temperature $X_{MT}$ are the same for all samples within the same day/donor. Each β indicates the magnitude of the corresponding model term’s impact on the outcome.

As the experimental design required runs to be performed across multiple days/donors, the individual observations were not treated as independent, which was reflected in the modeling by having two variance components: $\varepsilon_{0}$ corresponding to the between-day/donor variance and ε—to the variance within each day and between samples from an individual donor.

Given the number of runs available, and the need for a sufficient number of degrees of freedom for estimating error terms, the maximum feasible polynomial was the full second-order one, as above. The primary aim of inferring reliable knowledge about the influence of cPPs on the outcome across their experimental ranges was then translated to the statistical objectives of obtaining (a) high-quality estimates of fixed model terms β and (b) high-precision prediction of the outcome values.

The statistical objectives were achieved by incorporating two optimality criteria (functions of the design) to search for the optimal design: (a) ‘’DP-criterion”, which minimizes the joint variance of the estimates of β [15,16], and (b) “IP-criterion”, which minimizes the prediction variance [17] and improves the quality of the predictions derived from the model. In addition, we accommodated the possibility that, had it been feasible, adding higher-order polynomial terms (of degree 3) to the model could have potentially provided a better fit for the data, especially as there is not enough prior information as to how the ‘true’ relationship is shaped. This was addressed by incorporating two more optimality criteria in the design search: (c) one that protected the inference on the model parameters β-s from this potential misspecification and (d) another that optimized the ability to test the model for the lack of fit with respect to higher-order terms [18]. Carrying out multi-objective optimization by combining these four individual criteria produced several designs, and the one that minimizes the joint variance of the model parameters’ estimates was found to perform well overall and hence was chosen to conduct the experiments (Table 2).

### 2.4. RAPID Gel Quality Attributes

Based on previous work [13], two principal outcomes were selected as indicators of manufacturing consistency and gel quality. The time to gel was recorded when the material changed from liquid to gel form following mixing of L-PRP with autologous thrombin. The exudation of releasate from the gel was determined by aspirating and measuring the mass of liquid exuded by gel at 15, 30, 45, 60 and 120 min and summing the cumulative release over 2 h. Thrombin clot time was also measured by recording the time it took to produce a visible clot inside the thrombinator device and as a putative in-process indicator of the final product (gel) quality.

### 2.5. Human Thrombin–Antithrombin Complexes

The variability in autologous thrombin produced was also quantified by measuring the thrombin–antithrombin (TAT) complex concentration using a human thrombin–antithrombin complex ELISA Kit (Abcam, Cambridge, UK). Autologous thrombin samples were frozen at the point of thrombin manufacture, and thawed, centrifuged and diluted according to the manufacturer’s instructions when analyzed.

### 2.6. Statistical Analysis

Data were analyzed using Microsoft Excel (version 16.102.3), R studio (version 2023.06.1) and Python (version 3.9.7). Modeling was performed in R (version 4.4.2) using the lme function from the nlme (v. 3.1.163) package [19], glmer function from the lme4 (v. 1.1.34) package [10] and coxme function from the coxme (v. 2.2.18.1) package [20].

## 3. Results and Discussion

### 3.1. Gel Manufacture

All donor blood was within accepted limits as outlined by the Haematology Reference Ranges [21] and full blood counts are provided in the Supplementary Data, Table 1. Blood processing using the Angel centrifuge resulted in a 0.66 reduction in white blood cell count and a 3.81-fold increase in platelet concentration. Notable differences were observed in extent to which centrifugation altered the WBC and platelet concentrations (Table 3).

**Figure 2:**
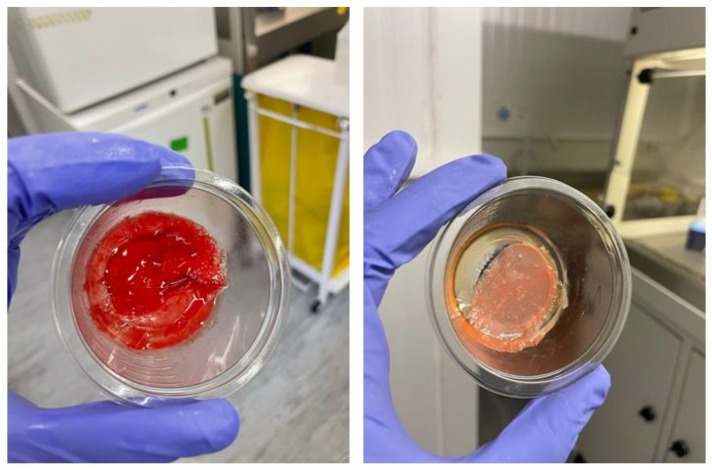
Illustrative RAPID gels made with two different Angel protocols with different hematocrit settings with 8% hematocrit on the left (acute protocol) and 2% hematocrit on the right (chronic protocol).

The manufacturing process of individual gels from the first centrifugation stage to gel formation took between 39 and 78 min. Four preparations did not gel within 10 min, which was designated a failure of the manufacturing process. The gels produced across the study weighed 9.0 ± 1.0 g (*n* = 28); time to gel was 181 ± 179 s and the mass of releasate exuded in 2 h was 5.4 ± 2.2 g. Visual differences were noted between the preparations with 2% (chronic protocol) vs. 8% (acute protocol) hematocrit protocols with color differences indicating variation in the level of red blood cell contamination (Figure 2). The chronic protocol (2% hematocrit) results in L-PRP having fewer red blood cells, thus appearing less red than the acute protocol.

As expected, the hematocrit (Hct) setting influenced the white blood cell (WBC) count in the L-PRP. The WBC levels increased from 2.72 × 10^6^ cells/mL at Hct 2% to a peak of 4.20 × 10^6^ at Hct 5%, before decreasing slightly to 3.61 × 10^6^ at Hct 8%. This indicates a non-linear relationship, with the highest WBC yield at the intermediate hematocrit setting.

### 3.2. Effects of Temperature and Pre-Mixing of L-PRP with Ascorbic Acid

**Figure 3:**
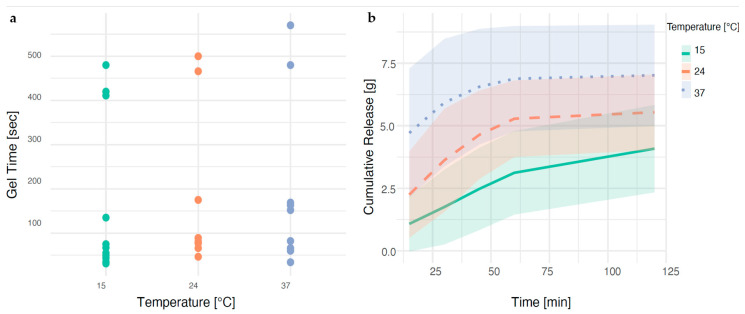
Effect of temperature on (**a**) time to gel, (**b**) releasate exudation. Data are grouped and plotted as a line graph with standard deviation shaded around the trendline (**b**). A portion of these data was previously presented as a conference poster at the 14th World Meeting on Pharmaceutics, Biopharmaceutics and Pharmaceutical Technology (Vienna, 2024) (Olszewska et al., 2024) [22].

**Figure 4:**
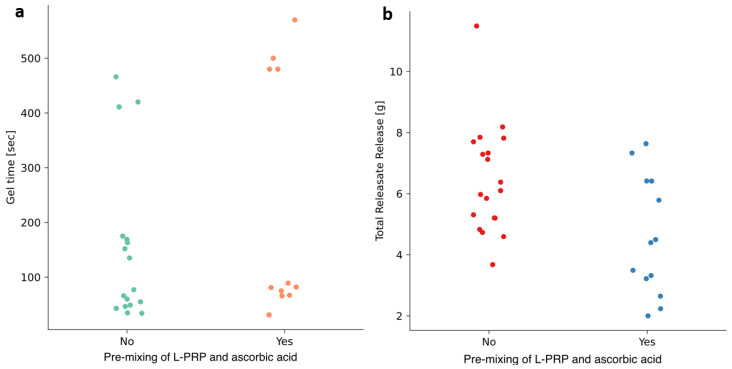
Effect of pre-mixing of L-PRP and ascorbic acid on (**a**) time to gel and the (**b**) mass of releasate exuded in 2 h. Each point represents an individual experimental run; groups represent samples either pre-mixed with ascorbic acid (Yes) or not (No). Colors are used for visual distinction only and do not indicate additional variables.

Of the variables investigated, the temperature and pre-mixing of L-PRP with ascorbic acid had readily apparent individual effects on the mass of releasate exuded, but not on the time to gel (Figure 3 and Figure 4). The other variables, mixing time, hematocrit setting, time-to-thrombin use and filtering of the thrombin, did not show such clear effects (Supplementary Data).

No relationship was observed between temperature and time to gel. Responses showed two clusters around longer gel times 400–500 s and gel times < 150 s. In contrast, the effect of temperature on the mass of releasate exuded (Figure 3a) was clear, with higher temperatures yielding greater exudation of release in 2 h. Releasate exuded from gels manufactured at 24 and 37 °C plateaued at around 60 min. In contrast, gels manufactured at 15 °C continued to exude releasate until the final measurement at 2 h. Additional data supporting these findings, including time-course release profiles and full correlation matrices, are provided in the Supplementary Materials (Figures S1–S4).

Pre-mixing of L-PRP with ascorbic acid did not appear to affect time to gel; the gels that took longer to form were distributed across the runs that did and did not have pre-mixing of these reagents (Figure 4a). Pre-mixing of L-PRP with ascorbic acid was negatively associated with the mass of releasate exuded in 2 h (Figure 4b).

### 3.3. Statistical Evaluation of Processing Parameter Influences and Interactions

Inference was drawn from the linear mixed model (Equation (1)) and was performed using the restricted maximum likelihood (REML) method for estimating the random effects (donor effect), and Generalized Least Squares (GLS) for the fixed effects (selected processing parameters) estimation [14]. The summary of fitting the simplified models for the exudation of releasate in 2 h and time to gel is presented in Table 4.

#### 3.3.1. Exudation of Releasate

A higher manufacturing temperature resulted in a greater exudation of releasate in 2 h, and that increase was larger for longer times of time-to-thrombin use—as per positive $\beta_{MT\times t}$ interaction term. Pre-mixing of L-PRP and ascorbic acid, however, had a negative additive effect on the amount of releasate exuded in 2 h, by approximately 1.69 units (grams). Overall, the model revealed that both temperature and pre-mixing significantly affect the total mass of releasate exuded, with a notable interaction effect between temperature and time-to-thrombin use.

**Figure 5:**
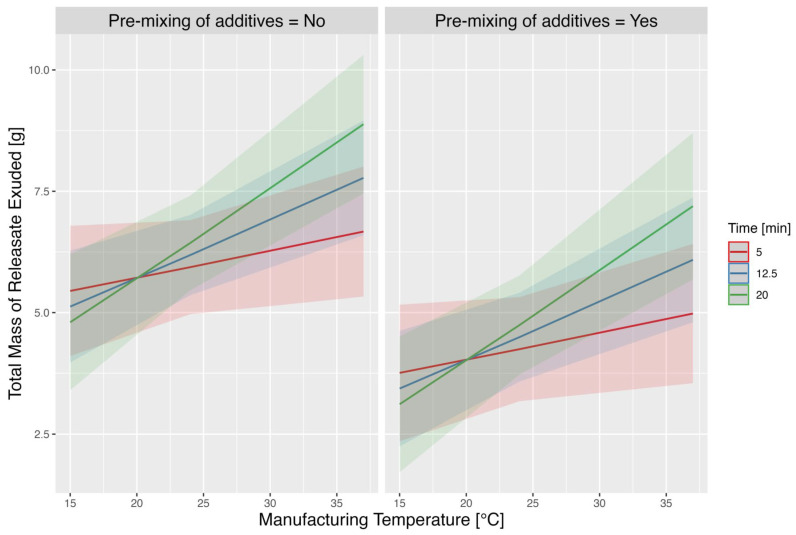
**Predicted total mass of releasate exuded in 2 h under different manufacturing temperature and mixing conditions.** The predicted total mass of releasate exuded across a range of manufacturing temperatures (15 °C to 37 °C) under two conditions: without pre-mixing of PRP and ascorbic acid (left panel) and with pre-mixing of PRP and ascorbic acid (right panel). The predictions are stratified by three different time points to thrombin use (5 min, 12.5 min and 20 min), represented by red, blue and green lines, respectively. Shaded areas around each line indicate the 95% confidence intervals for the predictions.

Graphical representation of this relationship can be seen in Figure 5, where the mean predicted response is displayed with 95% confidence interval bands for different values of the factors. Variance components were estimated at ${\hat{\sigma }}_{0}^{2}=0.62$ and ${\hat{\sigma }}^{2}$ =1.45, so that individual variability within each donor is roughly two times larger than between donors.

#### 3.3.2. Time to Gel

**Figure 6:**
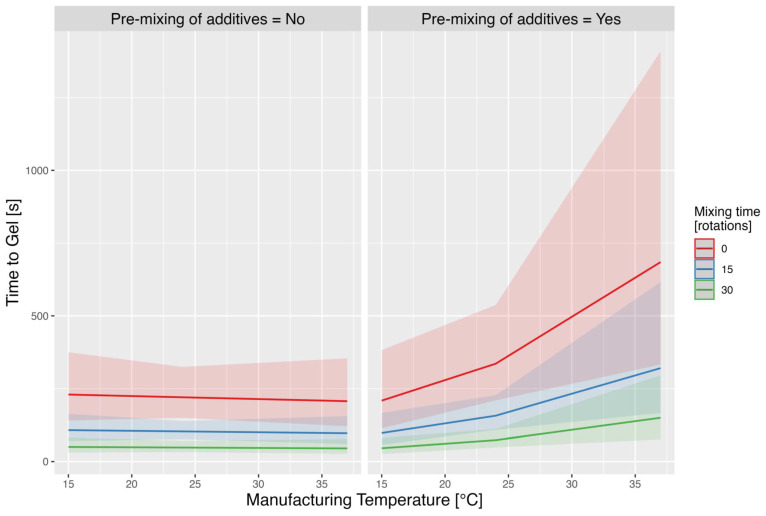
Predicted time to gel across the range of selected manufacturing temperatures and mixing rotations with or without pre-mixing of additives. This figure shows the predicted gelation time (in seconds) across a range of manufacturing temperatures (15 °C to 37 °C), with and without pre-mixing of additives. The left panel represents conditions where no pre-mixing of additives occurred, while the right panel shows the results when pre-mixing was performed. The predictions are further stratified by three different mixing times (0, 15 and 30 rotations), represented by red, blue and green lines, respectively. The shaded regions around each line represent the 95% confidence intervals for the predictions.

A log-transformation was used to fit a mixed-effects model similar to the model fitted for the exudation of releasate response. The increase in mixing time (from 0 to 15 rotations, and from 15 to 30 rotations) resulted in a reduction in the time to gel by the factor of $\exp\left(\beta_{T}\right)\;=\;0.468$, i.e., the gel was formed almost twice as fast (with all the other factors’ values remaining the same). Pre-mixing of L-PRP and ascorbic acid, however, led to an extension of the time to gel by the factor of $\exp\left(\beta_{M}\right)\;=\;1.73$ for the temperature of 26 °C, and even more up to the factor of $\exp\left(\beta_{MT}+\;\beta_{M}+\beta_{MT\times M}\right)\;=\;3.14$ for the highest temperature of 37 °C (Figure 6).

### 3.4. Discretized Response Modeling for ‘Successful’ Gel Formation

In clinical settings, it might be of interest to model the discretized “gel response” besides the time to gel. For example, the response could be set to 1 (‘success’) if gel has been formed in under 300 s, and 0 (‘failure’) otherwise, with the responses for the four experimental runs that resulted in no gel being formed also set to ‘failure’.

To predict a binary response (1/0 or success/failure) a logistic regression model [23,24], Equations (3) and (4), was fitted; it results in the inference summarized in Table 5. Increasing the mixing rotations increases the log-odds ratio of the gel forming within 300 s, with the expected probability of about 0.4 at 0 rotations, 0.7—at 15 rotations and 0.9—at 30 rotations.

(3) $$log\frac{p(x_{i})}{1-p(x_{i})}=\beta_{0}+\beta^{T}x_{i}+\varepsilon_{i}$$

(4) $$\hat{p}\left(x^{\prime }\right)\;=\;\frac{\exp\left(\beta_{0}\;+\;\beta^{T}x^{\prime }\right)}{\exp\left(\beta_{0}\;+\;\beta^{T}x^{\prime }\right)+\;1}$$

Logistic regression (3): $p\left(x_{i}\right)$—probability of gel formation of the i-th run of the experiment (i=1...n). The right-hand side is the standard regression $,\varepsilon_{i}$ are assumed to be independent normal with 0 mean and constant variance. The estimated probability of ‘success’, i.e., gel forming for a mixture with cPPs $x^{\prime },$ can be calculated as in (4).

The accuracy of the fitted model is 78.1%, the sensitivity is 76.2% and precision is 88.9%; with the F-score (the harmonic mean of the model’s recall and precision) being equal to 82%, the model provides a reliable performance in terms of identifying ‘success’ responses.

### 3.5. Time-to-Gel Analysis: Survival Modeling

**Figure 7:**
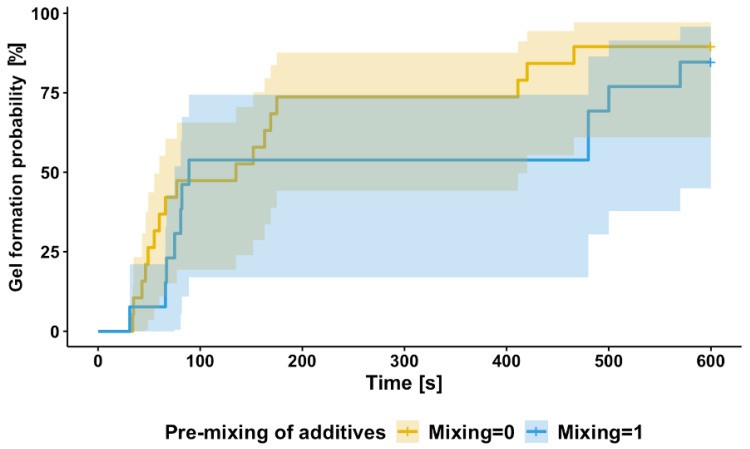
Probability of gel formation over time from platelet after thrombin activation when manufactured with or without pre-mixing of L-PRP and ascorbic acid. This figure illustrates the probability of gel formation over time following platelet activation with thrombin, stratified by the pre-mixing of L-PRP and ascorbic acid. The solid yellow line represents conditions without pre-mixing, while the dashed blue line corresponds to experiments where pre-mixing was applied. The shaded regions around each line represent the 95% confidence intervals for each condition.

Another approach to investigate the effect of cPPs on gel time is within a survival analysis framework that models the “time-to-event” responses, i.e., time to gel, which accounts for observations where no gel was formed through so-called censoring [25]. We start by looking at the probability of gel formation after time point t (in other words, the probability of no gel formation before time t), which is defined as the survival function S(t): it takes values between 0 and 1 and monotonically decreases with time. The probability of gel formation by time point t is then complementary to the survival function, 1−S(t), and its estimator for the data is illustrated in Figure 7: time-to-gel observations under the conditions with and without pre-mixing of L-PRP and ascorbic acid [26]. The graph shows a rapid increase in gel formation probability during the early time points with and without pre-mixing. However, pre-mixing delays the time to gelation, as seen by the lower probability for the pre-mixing condition at later time points. By 600 sec, both conditions approach similar probabilities of occurrence, although gel formation is more likely to occur earlier in the absence of pre-mixing.

A mixed-effects Cox model [23] was fitted to approximate the relationship between the factors (similar to the LME model (Equation (1)) and the response $\frac{h(t)}{h_{0(t)}}$—hazard ratio—ratio of gelation probabilities at time t, given that it has not occurred before, between the samples with baseline cPPs (all factors’ values equal to 0) and cPPs $x_{ij}$:

(5) $$\frac{h\left(t,x_{ij}\right)}{h_{0}\left(t\right)}=\exp\left(\beta^{T}x_{ij}+\gamma_{i}+\varepsilon_{ij}\right)$$

i=1…8; j=1…4.

Here, $\gamma_{i}$ and $\varepsilon_{ij}$ denote the between- and within-donor error terms, and $\beta^{T}x_{ij}$—fixed/polynomial part of the model; a unit increase in a predictor variable results in an exp(β) increase in the probability of gelation.

The summary of the final fitted model is given in Table 6 below; an increase in manufacturing temperature, time-to-thrombin use as well as pre-mixing of the initial additives leads to a decreased probability of gelation and, therefore, increased probability of later gel times. The effect of mixing rotations is opposite: mixing for longer increases the chance of gel forming sooner. The findings of this model agree with the results of the mixed-effects model in Table 4b and logistic regression summarized in Table 5. The between-donor variance is estimated at 0.074 and within-donor—at 0.0003, indicating that most of the variability across observations occurs due to the variability across individual donors. Full model diagnostics and variance component estimates are provided in the Supplementary Materials, (Tables S2–S4).

### 3.6. Effect of Temperature on Thrombin–Antithrombin Complexes and Thrombin Clot Time

**Figure 8:**
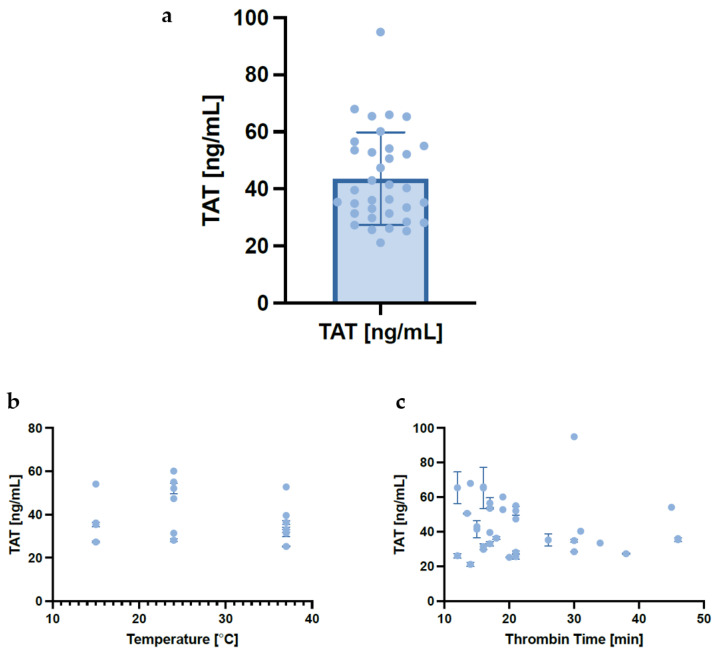
**Concentrations of thrombin–antithrombin (TAT) complex across experimental conditions.** Values were interpolated from a standard curve and are presented as mean ± sd. (**a**): A bar plot shows the overall distribution of TAT concentrations across all experimental runs. (**b**): A scatter plot illustrating TAT concentrations as a function of manufacturing temperature (°C). (**c**): A scatter plot showing TAT concentrations as a function of thrombin time (minutes). Abbreviations: TAT—thrombin–antithrombin complex.

As well as gel product attributes, two measures of in-process variability were made as follows: quantification of thrombin–antithrombin (TAT) complexes and thrombin clot time. In order to understand the variability in the amount of autologous thrombin being generated in the thrombinator reaction, thrombin–antithrombin (TAT) complexes were quantified. A notable variety was observed not only across donors but also across different runs (Figure 8).

**Figure 9:**
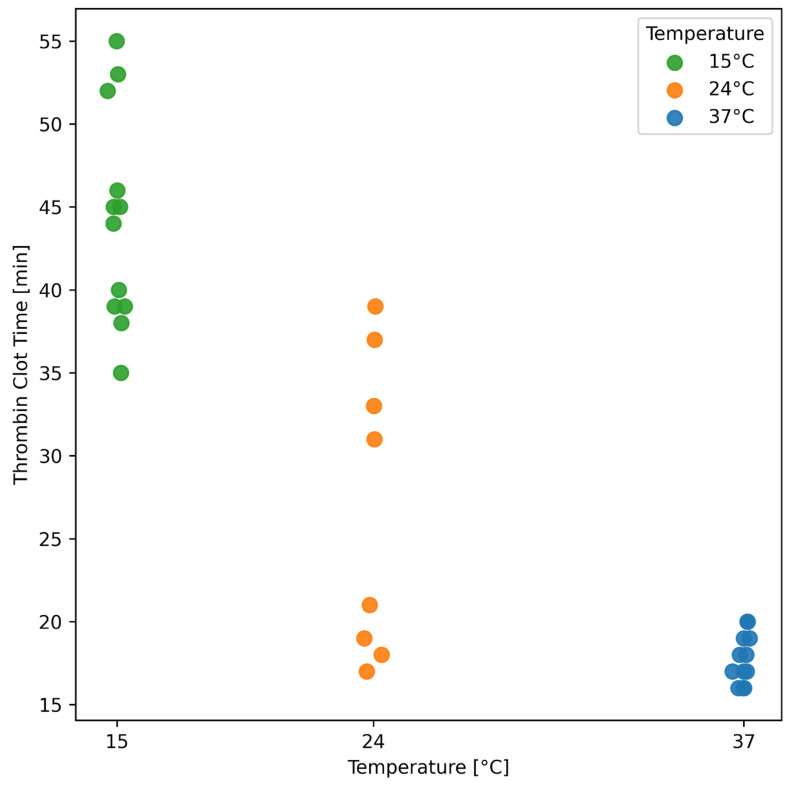
The effects of manufacturing temperature on thrombin clot time. Data represent individual measurements.

No trends were observed between the temperature and detected TAT complexes’ concentration nor between detected thrombin time and TAT concentration; as other variables were downstream of this process, they would not be expected to have an effect, therefore, were not tested. As expected, temperature affected the thrombin clot time (Figure 9) with the shortest clotting times and lower variability in thrombin clot time being observed at the highest temperatures.

## 4. Discussion

The RAPID Biodynamic Haematogel point-of-care manufacturing process has been reported previously [13]. In this study, we applied a design of experiments methodology to explore which elements of the manufacturing process of RAPID require controlling to produce the gel reproducibly by investigating six processing parameters: four continuous and two binary ones. The results confirmed that the process has inherent variability as might be expected of an autologous blood product due to differences between donor blood, which serves as the starting material for L-PRP gel manufacture. The manufacturing process uses volumetric measurements throughout and does not control for the quantity of platelets or other cells in the final product, i.e., beginning with 60 mL blood and diluting the L-PRP to 6 mL as a manufacturing step (Figure 1). We observed variability in cell counts across the eight donors whose blood was used in this study, with a coefficient of variation of 73.5% for white blood cell counts and 68.2% for platelet counts in the L-PRP used to form the gels. Statistical analysis confirmed the impact of difference across individual donors on the probability of gelation, with an estimated inter-donor variability exceeding the within-donor one by almost 200 times (Supplementary Material, Table 4). A level of red blood cell contamination was observed in all the gels, with 8% hematocrit RAPID gels having the strongest red color. Red blood cell content needs to be further investigated as it has been shown that red blood cells may release free iron when damaged, which might have a negative impact due to the presence of potent pro-inflammatory cytokines, which are activated when iron among other molecules is released from damaged RBCs.

Differences were also observed in autologous thrombin formation, as indicated by the thrombin–antithrombin complexes, with a range of 34.9 ng/mL (coefficient of variation of 28.4%), leading to variations in the time required to form a thrombin clot within the thrombinator device. This shows that there are even further intra-donor differences relating to a crucial component of the manufacturing process, the potent platelet activator—thrombin. However, no noteworthy relationships were found between TAT complexes and other variables, indicating that potentially the amount of thrombin that forms is supraoptimal and as long as a minimum level of thrombin activity is reached, the variability does not have a notable effect on gel time nor is it influenced by thrombin time as noted here. Further work needs to be conducted to better understand these relationships.

Of the processing variables, the manufacturing temperature notably influenced thrombin formation, with higher temperatures leading to shorter thrombin clot formation times. This variability influenced the total manufacturing time, which ranged from 39 to 78 min. It is also important to note that this study only utilized healthy donor blood—future experiments could be conducted on blood from diabetic patients to make the results more relevant to the target patient population.

### 4.1. Time to Gel Is Affected by Mixing During Manufacture

When time to gel was used as a key product attribute, it was significantly reduced by pre-mixing ascorbic acid with L-PRP before combining with thrombin-rich serum. Furthermore, the extent of mixing after combining L-PRP with serum, measured in mixing rotations, had a direct correlation with time to gel, with greater mixing leading to shorter gel times. This is due to multiple factors starting from an even distribution of all necessary components of the clotting reaction. The shear stress of mixing may lead to accelerated platelet activation even before thrombin is added; lastly, as this reaction is based on fibrin polymerization, the even distribution of fibrinogen as part of the pre-mixing of L-PRP with ascorbic acid leads to a more uniform distribution of a structural framework that eventually forms the clot, the L-PRP RAPID gel [27,28].

Given the discrete nature of the time-to-gel response, additional analysis was performed by classifying gel times shorter than 300 s as a ‘product pass’ and longer times as a ‘product fail’. This binary classification allowed us to better understand the impact of the processing parameters on gel success rates. We observed that an increased mixing time increased the likelihood of achieving a ‘successful’ gel time, while pre-mixing decreased the chances of gelation at any point and prolonged the expected time it would take the mixture to gel, especially for higher temperatures.

These findings indicate that time to gel could be a quality attribute for control for point-of-care manufacturing where quality control and assurance is tricky. Shorter gel times might reflect higher thrombin levels in the autologous thrombin serum generated, a component of the RAPID manufacturing process. Gel time is also likely to be indirectly influenced by factors such as the prothrombin and fibrinogen concentration in blood [29,30] as well as platelet activation—all of which are challenging to control. Future investigations into the quality of RAPID gels should include the relationship between time to gel and fibrinogen levels and the impact of intrinsic factors compared to manufacturing variables on time to gel.

### 4.2. Exudation of Releasate Is Affected by Manufacturing Temperature and Mixing

When considering the exudation of releasate from the gel in 2 h as a quality attribute, the linear mixed-effects model revealed that the manufacturing temperature and pre-mixing of ascorbic acid with L-PRP significantly influenced the exudation of release from RAPID gels. It has previously been shown that more extreme, high temperatures may lead to protein denaturation and faster gel breakdown [31,32], as well as that cooling PRP slows enzymatic activity and allows gel stability for extended periods of time; temperature has also been linked to enzyme kinetics and a more sustained release of growth factors at lower temperatures [33], which is similar to what we observed with liquid exudation from RAPID gels.

Higher manufacturing temperatures and the absence of pre-mixing resulted in a greater mass of releasate exuded in 2 h. Notably, the lowest tested temperature, 15 °C, was associated with the slower exudation of liquid from the gel (Figure 3b). This suggests the potential for a sustained exudation profile at lower temperatures, which could be utilized strategically in product development to tailor the release kinetics according to therapeutic needs.

Temperature also had a pronounced effect on the thrombin clot time, which is a critical step in the manufacturing process. We observed that higher temperatures, particularly 37 °C, resulted in the quickest and most consistent thrombin clot times. This aligns with established knowledge that 37 °C is the optimal temperature for thrombin clotting and activity [34]; while we did not see any apparent trends between thrombin–antithrombin complex generation at various temperatures and we have not measured the thrombin activity directly, previous studies have reported the role of lower temperatures on lower thrombin activity [34,35]. Interestingly, thrombin clot times at 15 °C were significantly longer, extending up to 55 min compared to just 16 min at 37 °C. Given that this step heavily influences the overall manufacturing time, adjusting the protocol to maintain a consistent temperature of 37 °C could minimize variability and enhance process efficiency, balanced with the need for consistent release kinetics.

However, it is crucial to consider that the behavior of these gels after manufacture will differ in a wound environment, where inflammation may elevate temperatures above normal skin temperature. This might result in faster exudation from the gel, underscoring the need for in vivo or ex vivo studies to further elucidate the impact of wound conditions on releasate dynamics. Previous studies conducted on similar preparations indicated different release profiles in vitro and ex vivo [36].

### 4.3. Design of Experiments Approach Is a Cost-Effective and Efficient Methodology for Pharmaceutical Product Development and Design

This study used a design of experiments approach in order to address the study questions, while using human blood as optimally as possible. DoE extracts the maximum information from a limited number of runs and is extremely beneficial when working with a limited resource such as human blood. DoE methodology has long been established in pharmaceutical development, but it has not been widely used in the development of biologic medicinal products [9]. With autologous products and POC manufacture, where quality control often proves to be tricky, DoE approaches might be particularly useful in identifying critical processing parameters quickly and efficiently.

This study demonstrated how DoE can optimize resource use in pharmaceutical research and development. By reducing the experimental burden, DoE can significantly shorten development timelines, reduce costs and enhance the overall quality of the final product. As the pharmaceutical industry increasingly moves towards personalized medicine and complex biologics, the role of DoE in streamlining development and ensuring product efficacy and safety will likely become even more important.

### 4.4. RAPID Gel Manufacturing Process

The literature reports a number of different preparation methods for PRP and other platelet-based therapies, and there is a growing consensus on the need for some degree of standardization, for example, agreed-upon ranges of platelet counts necessary for therapeutic effectiveness [37,38]. Future work in developing RAPID gel as a medicinal product could include controlling the number of platelets or white blood cells, both of which may impact the therapeutic efficacy of the final product [39]. For the RAPID gel, control of the platelet number could be achieved by setting a narrow working range for the platelet concentration in L-PRP and diluting the L-PRP to this concentration during the relevant manufacturing stage (Figure 1).

Variability in time to gel may be reduced by tightening the control of the thrombin levels generated during manufacturing, as thrombin directly activates the coagulation cascade and fibrin matrix formation [40], both of which are essential for gel formation. Alternatively, it is possible that the thrombin levels generated and used are supraoptimal, and as long as it is used promptly, there is little effect on time to gel. Generating thrombin at 37 °C, which is the physiological temperature for thrombin generation, could also be adopted to reduce product variability. Establishing the optimal amount of thrombin for effective platelet activation and gel formation and determining how process parameters affect this are also questions for ongoing product development. The overarching goal is to control the critical processing parameters of the manufacturing process and assure product quality within the constraints of the POC manufacture of an autologous product at the patient’s bedside.

An essential consideration in the development of a standardized point-of-care (POC) manufacturing process is the practicality of product manufacture and usability in clinical settings. When designing such processes, an important consideration is the capability of healthcare providers who may not have specialized training in laboratory techniques or access to laboratory equipment. Therefore, the process needs to be simple and robust such that it can be executed reliably in a wide range of environments. For example, while it might be advantageous to tightly control the temperature during the generation of autologous thrombin given its significant impact on the duration and consistency of the manufacturing process, it could be challenging to achieve in a POC setting. Simple, reliable monitorable key process variables, such as ‘acceptable’ gel times that would indicate a high level of thrombin [41] and acceptable level of platelet activity/activation [42], could help bridge the gap between laboratory precision and bedside practicality.

## 5. Conclusions

This study focused on the point-of-care (POC) manufacturing protocol for the autologous RAPID™ Biodynamic Haematogel. The optimization and application of a bespoke design of experiments (DoE) methodology enabled six processing variables to be systematically investigated while accounting for constraints in the randomization of conditions. This demonstrates the utility of statistical experimental design in the efficient development of complex biologic pharmaceutical products and supports the adoption of a quality by design approach to product development. For the RAPID gel, the study identified the manufacturing temperature and mixing as putative critical processing parameters to improve consistency in time-to-gel formation and releasate exudation after manufacture. Other aspects that might benefit from further standardization include the platelet and thrombin concentrations. However, any measures to improve product quality must be harmonized with the practicalities of POC manufacturing in clinical settings, where simplicity and usability remain paramount. These findings provide a foundation for further refinement of manufacturing parameters and will inform future studies aimed at evaluating clinical performance and scalability.

## Figures and Tables

**Table 1:** **Process parameters as experimental factors used in the linear mixed-effects model equation.** Six process parameters were used in the design of experiments with a selected range of values; four continuous process parameters were tested at three levels within a selected range; two binary process parameters were tested at two levels, as denoted below. Scaled levels as well as the equation factor corresponding to each process parameter are shown in the table.

Factor	Process Parameter	Range of Values	Levels	Scaled Levels
$X_{MT}$	Temperature [°C]	15–37 °C	{15, 24, 37}	{−1, −0.18, +1}
$X_{T}$	Mixing time [rotations]	0–30 rotations	{0, 15, 30}	{−1, 0, +1}
$X_{W}$	WBC content [%]	2–8%	{2, 5, 8}	{−1, 0, +1}
$X_{t}$	Time-to-thrombin use [min]	5–20 min	{5, 12.5, 20}	{−1, 0, +1}
$X_{FT}$	Filtration of thrombin	No, Yes	{0, 1}	{−1, +1}
$X_{M}$	Pre-mixing of additives	No, Yes	{0, 1}	{−1, +1}

**Table 2:** Design of experiments matrix showing the processing conditions applied in each experimental run.

Day/Donor	Run	Temperature [°C]	Mixing Time [Rotations]	HCT [%]	Time-to-Thrombin Use [min]	Filtering of Thrombin	Pre-Mixing Ascorbic Acid/L-PRP
**1**	1	15	15	8	20	No	No
**1**	2	15	30	8	5	Yes	No
**1**	3	15	0	2	5	No	No
**1**	4	15	0	5	20	Yes	Yes
**2**	5	37	0	5	20	Yes	No
**2**	6	37	30	2	20	Yes	No
**2**	7	37	0	8	5	Yes	No
**2**	8	37	30	2	5	No	No
**3**	9	37	0	2	20	Yes	No
**3**	10	37	30	8	20	Yes	No
**3**	11	37	0	5	20	No	No
**3**	12	37	30	8	5	Yes	No
**4**	13	15	30	2	20	No	Yes
**4**	14	15	30	2	5	Yes	No
**4**	15	15	15	8	12.5	Yes	No
**4**	16	15	30	8	5	No	No
**5**	17	15	30	5	12.5	No	No
**5**	18	15	0	2	5	Yes	No
**5**	19	15	0	8	5	Yes	No
**5**	20	15	0	2	20	Yes	No
**6**	21	24	30	8	20	Yes	No
**6**	22	24	15	2	20	Yes	Yes
**6**	23	24	0	5	20	No	No
**6**	24	24	0	8	20	No	Yes
**7**	25	24	0	2	12.5	Yes	No
**7**	26	24	30	8	20	Yes	Yes
**7**	27	24	30	2	20	No	No
**7**	28	24	15	5	5	No	Yes
**8**	29	37	30	8	5	Yes	No
**8**	30	37	0	8	5	No	No
**8**	31	37	0	2	5	Yes	No
**8**	32	37	0	8	20	Yes	No

Abbreviations: HCT—hematocrit.

**Table 3:** Summary statistics of white blood cell and platelet counts in whole blood and platelet-rich plasma in eight donors whose blood was used for the study. Abbreviations: WBC—white blood cells, L-PRP—leukocyte and platelet-rich plasma, PLT—platelets.

Parameter	Mean	STD	Min	Max
WBC	5.43 × 10^6^	1.50 × 10^6^	2.72 × 10^6^	8.14 × 10^6^
WBC IN L-PRP	3.32 × 10^6^	2.44 × 10^6^	9.38 × 10^5^	8.63 × 10^6^
INCREASE FROM BASAL	0.66	0.43	0.14	1.45
PLT	2.54 × 10^8^	4.34 × 10^7^	1.99 × 10^8^	3.20 × 10^8^
PLT IN L-PRP	1.03 × 10^9^	7.03 × 10^8^	4.40 × 10^8^	2.24 × 10^9^
INCREASE FROM BASAL	3.81	1.94	1.89	7.25

**Table 4:** **Linear mixed model for total releasate exuded in 2 h (a) and log time to gel (b), with Kenward–Roger correction.** This table presents the results of a linear mixed model evaluating the effects of temperature, mixing time, time-to-thrombin use and pre-mixing of additives on two dependent variables: log time to gel and total releasate exuded in 2 h. Interaction effects between temperature and time-to-thrombin use were also included in the model. The Kenward–Roger approximation was applied to adjust the degrees of freedom, ensuring more accurate estimates.

**(a)**					
**Parameter**	**Value**	**Standard Error**	**Degrees of Freedom**	**t-Value**	***p*-Value**
Intercept, $\beta_{0}$	6.4499	0.3957	8.8723	16.236	0.0000
Temperature, $\beta_{MT}$	1.3260	0.4057	5.8767	3.268	0.0176
Time-to-Thrombin use, $\beta_{t}$	0.3917	0.2470	24.2309	1.586	0.1257
Pre-mixing of additives, $\beta_{M}$	−1.6888	0.4611	22.9054	−3.662	0.0013
Temperature x Time-to-Thrombin use, $\beta_{MT\times t}$	0.7144	0.2650	21.8555	2.696	0.0133
**(b)**					
**Parameter**	**Value**	**Standard Error**	**Degrees of Freedom**	**t-Value**	***p*-Value**
Intercept, $\beta_{0}$	4.62513	0.14513	11.30670	31.868	0.0000
Temperature, $\beta_{MT}$	−0.05217	0.16551	10.65287	−0.315	0.7587
Mixing time, $\beta_{T}$	−0.75937	0.13010	22.71603	−5.837	0.0000
Pre-mixing of additives, $\beta_{M}$	0.55067	0.24328	22.06006	2.264	0.0338
Temperature x Pre-mixing of additives, $\beta_{MT\times M}$	0.64610	0.29638	22.92721	2.180	0.0398

**Table 5:** **Logistic regression for the binary time-to-gel response.** This table presents the results of a logistic regression model predicting the binary gel time response based on the intercept and mixing time.

Parameter	Value	Standard Error	z-Value	*p*-Value
Intercept, $\beta_{0}$	0.8869	0.4704	1.885	0.0594
Mixing time, $\beta_{T}$	1.2973	0.5107	2.540	0.0111

**Table 6:** **Cox mixed-effects model for gel time.** This table presents the results of a Cox mixed-effects model analyzing the impact of temperature, time-to-thrombin use, pre-mixing of additives, mixing time and their interaction on gel time.

Parameter	Value	Exp (Value)	Standard Error	z-Value	*p*-Value
Temperature, $\beta_{MT}$	−1.03690	0.35455	0.31834	−3.26	0.001
Time-to-Thrombin use, $\beta_{t}$	−0.61069	0.54298	0.2429487	−2.51	0.012
Pre-mixing of additives, $\beta_{M}$	−1.02978	0.35709	0.4541926	−2.27	0.023
Mixing time, $\beta_{T}$	1.42476	4.15684	0.3452564	4.13	0.000
Temperature x Time-to-Thrombin use, $\beta_{MT\times t}$	−0.81193	0.44400	0.2822142	−2.88	0.004

## Data Availability

The data presented in this study are available on request from the corresponding author due to ethical restrictions.

## Supplementary Materials

The following supporting information can be downloaded at: https://www.mdpi.com/article/10.3390/pharmaceutics17111482/s1, Table S1. Summarized whole blood counts for all blood donors; Figure S1. The cumulative exudation of releasate measured at various time points (20, 40, 60, 80, 100, and 120 min) under three different temperatures: 15 °C, 24 °C, and 37 °C; Figure S2. Histogram of gel time responses showing a bimodal distribution, with two distinct groups; Figure S3. Correlation Matrix and Pairwise Scatter Plots for Key Experimental Variables; Figure S4. A correlation matrix displaying the relationships between different variables involved in the gelation process; Table S2. Checking the Proportional Hazards (PH) assumption after fitting the Cox mixed effects model for various parameters: temperature ($\beta_{MT}$), pre-mixing of additives ($\beta_{M}$), mixing time ($\beta_{T}$) and interaction term between temperature and time ($\beta_{MT\times t}$). Table S3. Variance components for log-gel-time mixed-effects model (model 4b); Table S4. Variance components for mixed-effects Cox regression model (Table 6).

## References

[1] Guest J.F., Fuller G.W., Vowden P. (2020). Cohort Study Evaluating the Burden of Wounds to the UK’s National Health Service in 2017/2018: Update from 2012/2013. BMJ Open.

[2] Armstrong D.G., Boulton A.J.M., Bus S.A. (2017). Diabetic Foot Ulcers and Their Recurrence. N. Engl. J. Med..

[3] McDermott K., Fang M., Boulton A.J.M., Selvin E., Hicks C.W. (2022). Etiology, Epidemiology, and Disparities in the Burden of Diabetic Foot Ulcers. Diabetes Care.

[4] Wang S., Yang J., Zhao G., Liu R., Du Y., Cai Z., Luan J., Shen Y., Chen B. (2021). Current Applications of Platelet Gels in Wound Healing—A Review. Wound Repair Regen..

[5] ICH Q8 (R2) Pharmaceutical Development-Scientific Guideline|European Medicines Agency (EMA). https://www.ema.europa.eu/en/ich-q8-r2-pharmaceutical-development-scientific-guideline.

[6] Kumar V., Madsen T., Zhu H., Semple E. (2005). Stability of Human Thrombin Produced From 11 Ml of Plasma Using the Thrombin Processing Device. J. Extra. Corpor. Technol..

[7] Mihalko E., Brown A.C. (2020). Clot Structure and Implications for Bleeding and Thrombosis. Semin. Thromb. Hemost..

[8] Fukuda I.M., Pinto C.F.F., Moreira C.D.S., Saviano A.M., Lourenço F.R. (2018). Design of Experiments (DoE) Applied to Pharmaceutical and Analytical Quality by Design (QbD). Braz. J. Pharm. Sci..

[9] Politis S.N., Colombo P., Colombo G., Rekkas D.M. (2017). Design of Experiments (DoE) in Pharmaceutical Development. Drug Dev. Ind. Pharm..

[10] Otava M., Mylona K. (2024). Split-Plot Experiments with Replicated Runs in Pharmaceutical Synthesis. Qual. Reliab. Eng. Int..

[11] Oberleitner T., Zahel T., Kunzelmann M., Thoma J., Herwig C. (2023). Incorporating Random Effects in Biopharmaceutical Control Strategies. AAPS Open.

[12] Diaz F.J., Yeh H.-W., de Leon J. (2012). Role of Statistical Random-Effects Linear Models in Personalized Medicine. Curr. Pharmacogenomics Pers. Med..

[13] Olszewska A., Duan J., Javorovic J., Chan K.L.A., Rickard J., Pitchford S., Forbes B. (2024). Manufacture and Initial Characterisation of RAPIDTM Biodynamic Haematogel, an Autologous Platelet and Leukocyte-Rich Plasma Gel for Diabetic Foot Ulcers. Gels.

[14] Letsinger J.D., Myers R.H., Lentner M. (1996). Response Surface Methods for Bi-Randomization Structures. J. Qual. Technol..

[15] Gilmour S.G., Trinca L.A. (2012). Optimum Design of Experiments for Statistical Inference. J. R. Stat. Soc. Ser. C Appl. Stat..

[16] Trinca L.A., Gilmour S.G. (2015). Improved Split-Plot and Multistratum Designs. Technometrics.

[17] de Oliveira H.M., de Oliveira C.B.A., Gilmour S.G., Trinca L.A. (2022). Compound Optimality Criteria and Graphical Tools for Designs for Prediction. Qual. Reliab. Eng. Int..

[18] Egorova O., Gilmour S.G. (2025). Optimal Response Surface Designs for Detection and Minimization of Model Contamination. Technometrics.

[19] Bates D., Mächler M., Bolker B., Walker S. (2015). Fitting Linear Mixed-Effects Models Using Lme4. J. Stat. Softw..

[20] Terry M. (2022). Therneau Coxme: Mixed Effects Cox Models. CRAN Repos..

[21] Haematology Reference Ranges. https://www.gloshospitals.nhs.uk/our-services/services-we-offer/pathology/haematology/haematology-reference-ranges/.

[22] Olszewska A., Egorova O., Gaggia G., Mylona K., Pitchford S., Rickard J., Forbes B. Controlling Point-of-Care Manufacture of an Autologous Platelet-Based Wound Healing Gel. Proceedings of the 14th World Meeting on Pharmaceutics, Biopharmaceutics and Pharmaceutical Technology (PBP).

[23] Cox D.R. (1958). The Regression Analysis of Binary Sequences. J. R. Stat. Soc. Ser. B Methodol..

[24] Hosmer D.W., Lemeshow S., Sturdivant R.X. (2013). Applied Logistic Regression; Wiley Series in Probability and Statistics.

[25] Clark T.G., Bradburn M.J., Love S.B., Altman D.G. (2003). Survival Analysis Part I: Basic Concepts and First Analyses. Br. J. Cancer.

[26] Kenward M.G., Roger J.H. (1997). Small Sample Inference for Fixed Effects from Restricted Maximum Likelihood. Biometrics.

[27] Dohan Ehrenfest D.M., Rasmusson L., Albrektsson T. (2009). Classification of Platelet Concentrates: From Pure Platelet-Rich Plasma (P-PRP) to Leucocyte- and Platelet-Rich Fibrin (L-PRF). Trends Biotechnol..

[28] Godoi T.T.F., Rodrigues B.L., Huber S.C., Santana M.H.A., Fonseca L.F.d., Santos G.S., Azzini G.O.M., Mosaner T., Paulus-Romero C., Lana J.F.S.D. (2022). Platelet-Rich Plasma Gel Matrix (PRP-GM): Description of a New Technique. Bioengineering.

[29] Risman R.A., Belcher H.A., Ramanujam R.K., Weisel J.W., Hudson N.E., Tutwiler V. (2024). Comprehensive Analysis of the Role of Fibrinogen and Thrombin in Clot Formation and Structure for Plasma and Purified Fibrinogen. Biomolecules.

[30] Burnouf T. (2013). Platelet Gels. ISBT Sci. Ser..

[31] Chen J.-L., Cheng W.-J., Chen C.-C., Huang S.-C., Chen C.P.C., Suputtitada A. (2022). Thermal Oscillation Changes the Liquid-Form Autologous Platelet-Rich Plasma into Paste-Like Form. BioMed Res. Int..

[32] Carmona J.U., López C. (2024). Effects of Temperature and Time on the Denaturation of Transforming Growth Factor Beta-1 and Cytokines from Bovine Platelet-Rich Gel Supernatants. Gels.

[33] Etulain J., Mena H.A., Meiss R.P., Frechtel G., Gutt S., Negrotto S., Schattner M. (2018). An Optimised Protocol for Platelet-Rich Plasma Preparation to Improve Its Angiogenic and Regenerative Properties. Sci. Rep..

[34] Whelihan M.F., Kiankhooy A., Brummel-Ziedins K.E. (2014). Thrombin Generation and Fibrin Clot Formation under Hypothermic Conditions: An in Vitro Evaluation of Tissue Factor Initiated Whole Blood Coagulation. J. Crit. Care.

[35] Mitrophanov A.Y., Rosendaal F.R., Reifman J. (2013). Computational Analysis of the Effects of Reduced Temperature on Thrombin Generation: The Contributions of Hypothermia to Coagulopathy. Anesth. Analg..

[36] Wang X., Fok M.R., Pelekos G., Jin L., Tonetti M.S. (2022). In Vitro and Ex Vivo Kinetic Release Profile of Growth Factors and Cytokines from Leucocyte- and Platelet-Rich Fibrin (L-PRF) Preparations. Cells.

[37] Chahla J., Cinque M.E., Piuzzi N.S., Mannava S., Geeslin A.G., Murray I.R., Dornan G.J., Muschler G.F., LaPrade R.F. (2017). A Call for Standardization in Platelet-Rich Plasma Preparation Protocols and Composition Reporting: A Systematic Review of the Clinical Orthopaedic Literature. JBJS.

[38] Fadadu P.P., Mazzola A.J., Hunter C.W., Davis T.T. (2019). Review of Concentration Yields in Commercially Available Platelet-Rich Plasma (PRP) Systems: A Call for PRP Standardization. Reg. Anesth. Pain Med..

[39] Lana J.F., Huber S.C., Purita J., Tambeli C.H., Santos G.S., Paulus C., Annichino-Bizzacchi J.M. (2019). Leukocyte-Rich PRP versus Leukocyte-Poor PRP-The Role of Monocyte/Macrophage Function in the Healing Cascade. J. Clin. Orthop. Trauma.

[40] Al-Amer O.M. (2022). The Role of Thrombin in Haemostasis. Blood Coagul. Fibrinolysis.

[41] Tarandovskiy I.D., Surov S.S., Parunov L.A., Liang Y., Jankowski W., Sauna Z.E., Ovanesov M.V. (2024). Investigation of Thrombin Concentration at the Time of Clot Formation in Simultaneous Thrombin and Fibrin Generation Assays. Sci. Rep..

[42] Monroe D.M., Hoffman M., Roberts H.R. (2002). Platelets and Thrombin Generation. Arterioscler. Thromb. Vasc. Biol..

