# Supplementary material for: A Design of Experiments Approach to Identify Critical Processing Parameters for Manufacture of an Autologous Platelet Gel for Diabetic Foot Ulcer
Source: Pharmaceutics. 2025 Nov 17;17(11):1482. doi: 10.3390/pharmaceutics17111482 (PMC12655297; doi:10.3390/pharmaceutics17111482)
Supplement: Supplementary file 1 [file pharmaceutics-17-01482-s001.zip › pharmaceutics-3899307-supplementary.docx]

SUPPLEMENTARY DATA

**WHOLE BLOOD COUNTS**

**Table S1**. Summarised whole blood counts for all blood donors.

|  | **Mean** | **std** | **min** | **max** |
| --- | --- | --- | --- | --- |
| **WBC** | 5.43E+06 | 1.50E+06 | 2.72E+06 | 8.14E+06 |
| **PLT** | 2.54E+08 | 4.34E+07 | 1.99E+08 | 3.20E+08 |
| **Neut** | 2.99E+06 | 1.17E+06 | 1.11E+06 | 5.27E+06 |
| **Lymph** | 1.85E+06 | 2.77E+05 | 1.27E+06 | 2.17E+06 |
| **Mono** | 4.71E+05 | 2.02E+05 | 1.70E+05 | 8.00E+05 |
| **EO** | 9.50E+04 | 4.68E+04 | 2.00E+04 | 1.50E+05 |
| **Baso** | 2.63E+04 | 1.43E+04 | 1.00E+04 | 5.00E+04 |

**RESPONSES**

**OBSERVATIONS**


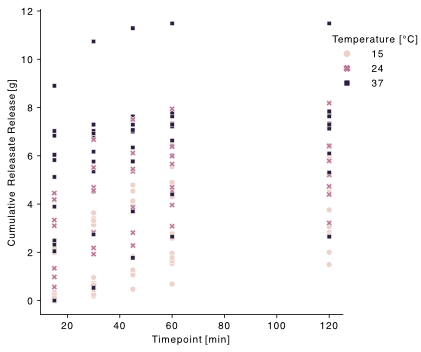


**Figure S1.** **The cumulative exudation of releasate measured at various time points (20, 40, 60, 80, 100, and 120 min) under three different temperatures: 15°C, 24°C, and 37°C.** The release profiles show the effect of temperature on the release rate, with higher temperatures (37°C) resulting in a greater cumulative release over time compared to lower temperatures (15°C and 24°C). Data points are represented as individual repeats and colour coded as beige circles (15°C), pint crosses (24°C), and navy squares (37°C). The y-axis represents the cumulative amount released in grams, and the x-axis represents the time in minutes.


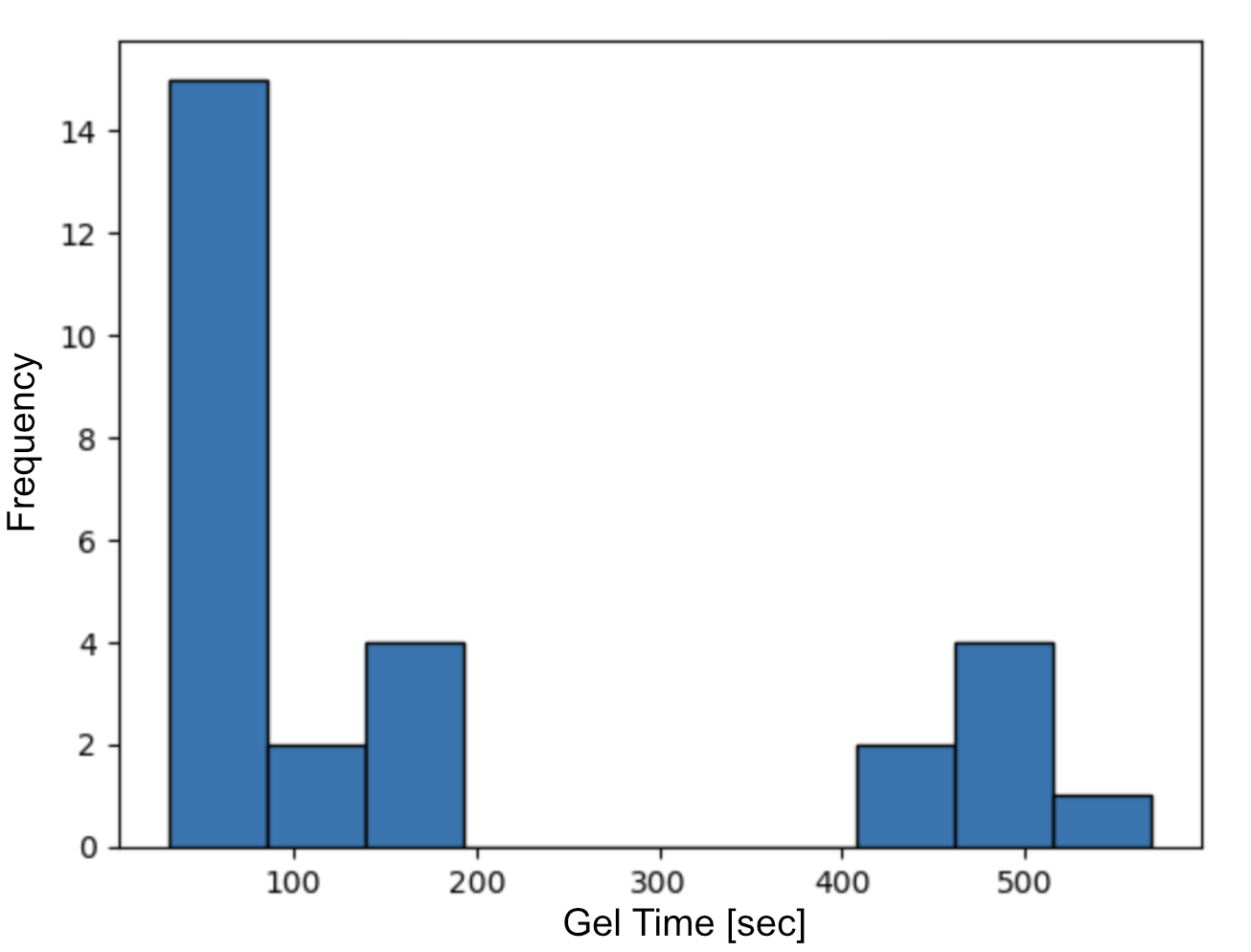


**Figure S2.** **Histogram of gel time responses showing a bimodal distribution, with two distinct groups**. The first group is concentrated around shorter gel times (approximately 100 seconds), while the second group centres around longer gel times (approximately 500 seconds).

**ANALYSIS AND INTERACTIONS**


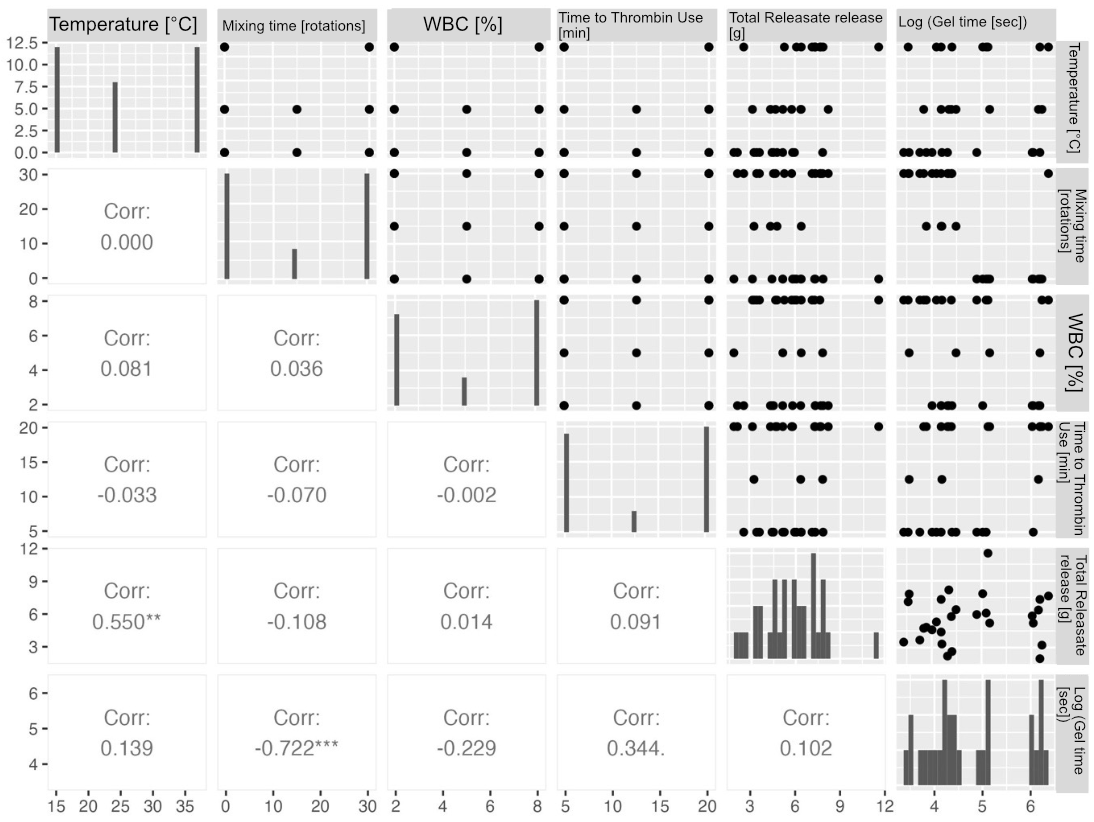


**Figure S3. Correlation Matrix and Pairwise Scatter Plots for Key Experimental Variables.** This figure displays a correlation matrix with pairwise scatter plots to visualize relationships between several key experimental variables: manufacturing temperature, mixing time, white blood cell (WBC), Time to thrombin use, total releasate release, and log-transformed gel time. **Upper diagonal panels:** Each plot represents the pairwise scatter plot between two variables, indicating the spread and distribution of values. **Lower diagonal panels:** Each cell contains the Pearson correlation coefficient between the corresponding pair of variables. Statistically significant correlations are indicated by asterisks (*p < 0.05, **p < 0.01, ***p < 0.001). The strongest negative correlation is observed between mixing time and log-transformed gel time (r = -0.722***), suggesting that increased mixing time significantly reduces gelation time. A significant positive correlation exists between temperature and total reaction rate (r = 0.550**), indicating that higher temperatures are associated with faster reaction rates.


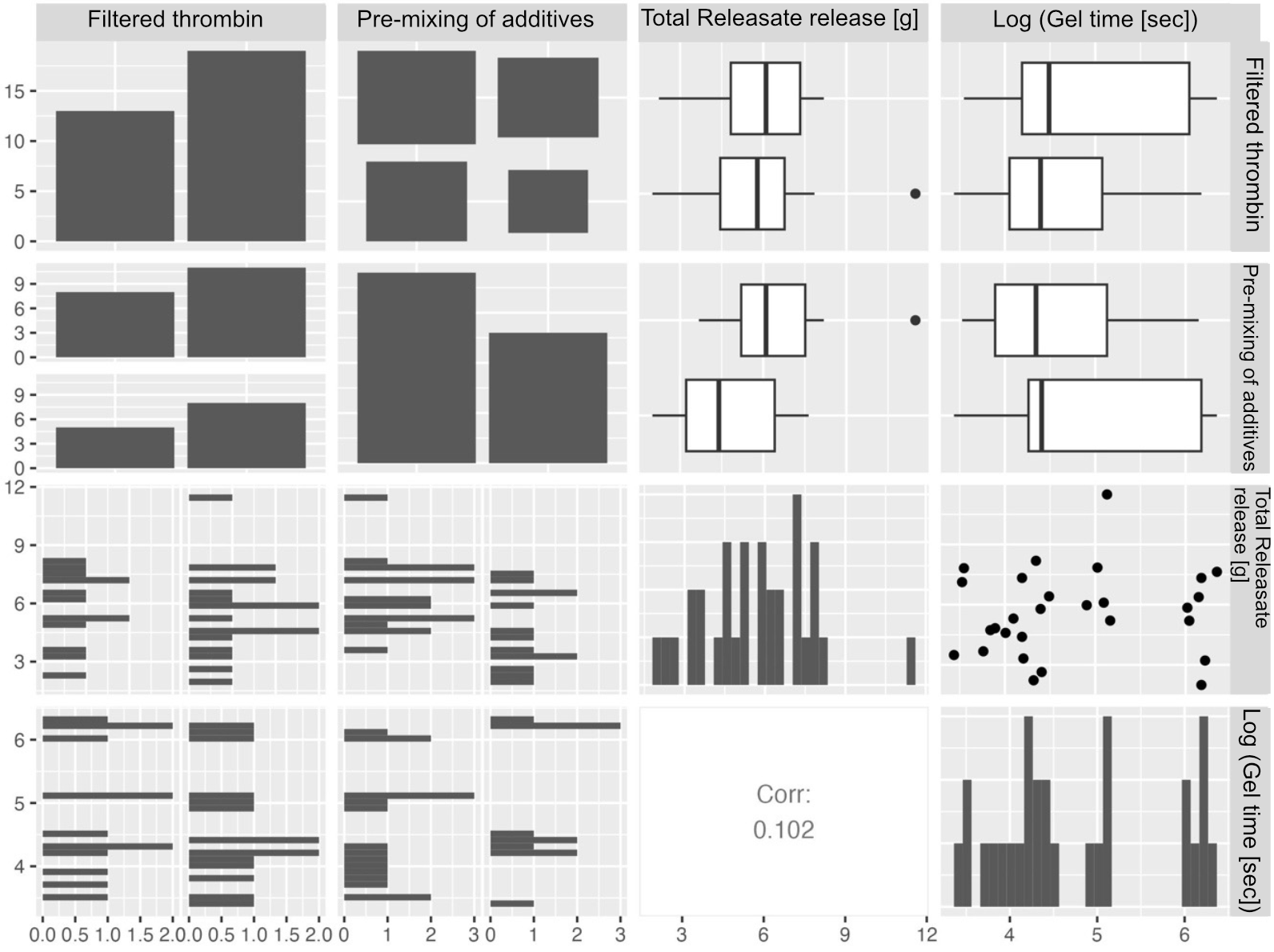


***Figure S4. A correlation matrix displaying the relationships between different variables involved in the gelation process.*** *Variables included in the matrix contain filtering of thrombin, pre-mixing of additives, total releasate release and log-transformed gel time. The diagonal panels show the histograms of each variable's distribution. The upper panels present box plots for each variable, providing a summary of their spread and any potential outliers. The lower panels depict scatter plots, illustrating pairwise relationships between the variables. The bottom-left cell displays the calculated correlation coefficient (Corr: 0.102) between "Total RR" and "Log (Gel time)," indicating a weak positive correlation.*

**MODELLING**

**Table S2. Checking the Proportional Hazards (PH) assumption after fitting the Cox mixed effects model for various parameters: temperature (**$\boldsymbol{\beta}_{\boldsymbol{MT}}$**), pre-mixing of additives (**$\boldsymbol{\beta}_{\boldsymbol{M}}$**) , mixing time (**$\boldsymbol{\beta}_{\boldsymbol{T}}$**) and** interaction term between temperature and time ($\beta_{MT\times t}$)**. The chi-square test statistic (chisq), and p-values for each parameter are provided.** An overall test was performed, showing the cumulative chi-square value and corresponding p-value. A p-value greater than 0.05 indicates that the PH assumption is not likely violated for the parameters tested.


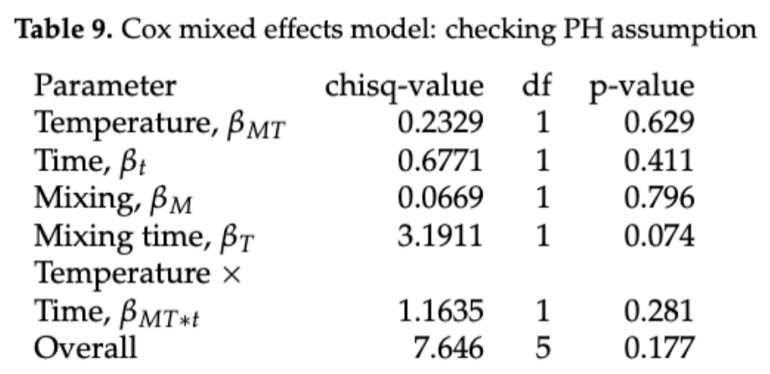


**Table S3. Variance components for log-gel-time mixed-effects model.** (model 4b). The model included a random intercept for Day. The estimated between-day variance was negligible (SD = 1.35 × 10⁻⁵), while the within-day residual variance was larger (SD = 0.584), indicating that most variability in log-gel-time is attributable to residual error rather than day-to-day differences.

# Random Effects

| Random Effect | Grouping Factor | Std. Dev. |
| --- | --- | --- |
| Intercept | Day | 1.347142e-05 |
| Residual | - | 0.5841782 |

**Table S4. Variance components for mixed-effects Cox regression model (Table 6).** The model included a random intercept for Day. Variance attributable to Day was much greater (0.0744) compared with Sample (0.00038), giving a variance ratio of ~194. This highlights that between-day variability was the dominant source of heterogeneity, as referenced in the Discussion.

# Random Effects

| Group | Variable | Std. Dev. | Variance |
| --- | --- | --- | --- |
| Day/Sample | (Intercept) | 0.0195674791 | 0.0003828862 |
| Day | (Intercept) | 0.2726921348 | 0.0743610004 |
